# Characterization of the relationship between FLI1 and immune infiltrate level in tumour immune microenvironment for breast cancer

**DOI:** 10.1111/jcmm.15205

**Published:** 2020-04-05

**Authors:** Shiyuan Wang, Yakun Wang, Chunlu Yu, Yiyin Cao, Yao Yu, Yi Pan, Dongqing Su, Qianzi Lu, Wuritu Yang, Yongchun Zuo, Lei Yang

**Affiliations:** ^1^ College of Bioinformatics Science and Technology Harbin Medical University Harbin China; ^2^ Public Health College Harbin Medical University Harbin China; ^3^ The State key Laboratory of Reproductive Regulation and Breeding of Grassland Livestock College of Life Sciences Inner Mongolia University Hohhot China

**Keywords:** breast cancer, FLI1, tumour immune microenvironment, tumour‐infiltrating lymphocyte

## Abstract

Breast cancer is the most common cancer and the leading cause of cancer death among women in the world. Tumour‐infiltrating lymphocytes were defined as the white blood cells left in the vasculature and localized in tumours. Recently, tumour‐infiltrating lymphocytes were found to be associated with good prognosis and response to immunotherapy in tumours. In this study, to examine the influence of FLI1 in immune system in breast cancer, we interrogated the relationship between the FLI1 expression levels with infiltration levels of 28 immune cell types. By splitting the breast cancer samples into high and low expression FLI1 subtypes, we found that the high expression FLI1 subtype was enriched in many immune cell types, and the up‐regulated differentially expressed genes between them were enriched in immune system processes, immune‐related KEGG pathways and biological processes. In addition, many important immune‐related features were found to be positively correlated with the FLI1 expression level. Furthermore, we found that the FLI1 was correlated with the immune‐related genes. Our findings may provide useful help for recognizing the relationship between tumour immune microenvironment and FLI1, and may unravel clinical outcomes and immunotherapy utility for FLI1 in breast cancer.

## 1 INTRODUCTION

Breast cancer is the most frequently diagnosed cancer and the leading cause of cancer death among women in the world.^1^ It is estimated that breast cancer accounted for 25% of newly diagnosed cancer cases and 15% of the cancer death in the world. In the United States, there are more than 270 000 newly diagnosed breast cancer patients and more than 40 000 new deaths due to this disease in 2018.^2^ Currently, different strategies, such as chemotherapy and radiotherapy are used to treat breast cancer. It is also effective for treating breast cancer by combination of different drugs, targeted therapy, hormone therapy, radiation therapy and surgery.^3^, ^4^, ^5^, ^6^ Despite the important advances in breast cancer therapy, the progress against breast cancer in the past years remains very slow.

Friend leukaemia virus integration 1 (FLI1) is a member of the ETS family, initially identified as a proto‐oncogene that highly expressed in retrovirus‐induced haematological tumours.^7^ It was shown that FLI1 was associated with the progression of tumour, served as a prognostic marker in many types of tumour and also acted as a potential therapeutic target in tumours.^8^, ^9^, ^10^, ^11^, ^12^ Many studies demonstrated that FLI1 was associated with autoimmunity and expressed highly in B cells and T cells during the lymphoid development.^13^, ^14^ However, the roles of FLI1 expression level in many types of cancers were only studied by a few researchers, and the results were often conflicted.^10^, ^15^, ^16^

Tumour‐infiltrating lymphocytes (TILs) were defined as the white blood cells left in the vasculature and localized in tumours.^17^, ^18^, ^19^ In recent years, many studies had recognized the importance of TILs in many types of tumours.^20^, ^21^, ^22^, ^23^ The immune system appears to influence the development of breast cancer.^24^, ^25^ In addition to these observations, the TIL was found to be associated with improved clinical outcomes in breast cancer.^24^, ^26^ Therefore, the biological functions and biological features of TILs were needed to be understood in the immune microenvironment of breast cancer.

In this study, the single sample gene set enrichment analysis (ssGSEA) was implemented to computationally infer the immune infiltration levels of 28 immune cell types in 1095 breast invasive carcinoma (BRCA) samples. Then, the association between the FLI1 expression level, immune infiltration levels of 28 immune cell types, cytolytic activity (CYT), tumour purity, ESTIMATE score, immune score, stromal score, leucocyte fraction, TIL regional fraction, lymphocyte infiltration (LI) signature score and immunomodulators were investigated, and significant correlations were found between them. Next, we characterized the immune infiltration patterns in high and low expression FLI1 subtypes of BRCA patients by using the immune infiltration levels of 28 immune cell types, immune‐related features and expression level of immunomodulators such as PD‐1, PD‐L1 and CTLA‐4. Furthermore, we confirmed that all FLI1 expression level was associated with the expression level of immune‐related genes by weighted gene co‐expression network analysis (WGCNA), like other immune‐related features, such as CYT and immune score. In addition, we evaluated the association of the FLI1 expression level with survival time. We believed that this integrative study substantially improved our understanding of the important role of FLI1 in tumour microenvironment in BRCA patients and established an approach that can easily be extended to other types of tumours in the future work.

As demonstrated by a series of recent publications ^27^, ^28^ and summarized in three comprehensive review papers,^29^, ^30^, ^31^ to develop a really useful predictor for a biological system, one needs to follow ‘Chou's 5‐step rule’^30^, ^31^, ^32^, ^33^, ^34^, ^35^, ^36^, ^37^, ^38^, ^39^ to go through the following five steps: (a) select or construct a valid benchmark data set to train and test the predictor; (b) represent the samples with an effective formulation that can truly reflect their intrinsic correlation with the target to be predicted; (c) introduce or develop a powerful algorithm to conduct the prediction; (d) properly perform cross‐validation tests to objectively evaluate the anticipated prediction accuracy; and (e) establish a user‐friendly web server for the predictor that is accessible to the public. Papers presented for developing a new sequence‐analysing method or statistical predictor by observing the guidelines of Chou's 5‐step rules have the following notable merits: (a) crystal clear in logic development, (b) completely transparent in operation, (c) easily to repeat the reported results by other investigators, (d) with high potential in stimulating other sequence‐analysing methods and (e) very convenient to be used by the majority of experimental scientists.

## 2 MATERIALS AND METHODS

### 2.1 Data set

The normalized gene‐level RNA‐Seq data of 1095 BRCA samples were retrieved from TCGA tumour samples (data accessed at GEO: GSE62944). The normalized gene‐level RNA‐Seq data of 113 normal patient samples for BRCA were also downloaded from the GEO data set (GSE62944).^40^ The values of overall survival and overall survival time for BRCA were obtained from the work of Liu et al.^41^ The immune‐related data set and molecular subtype information of BRCA were obtained from the supplementary files of Thorsson et al ^42^ and the manuscript page in Genomic Data Commons, which was available at the publication page (https://gdc.cancer.gov/about-data/publications/panimmune). The read count data of 1095 BRCA samples were also retrieved from TCGA tumour samples (data accessed at GEO: GSE62944).

### 2.2 Gene signatures and infiltration signatures

782 marker genes for 28 immune cell types were downloaded from the work of Charoentong et al.^43^ A list of angiogenesis genes was extracted from the work of Masiero et al,^44^ and a list of immunomodulator genes were extracted from the work of Thorsson et al.^42^ The CYT index, which was used to assess the intratumoural immune cytolytic T cell activity in tumours, was calculated as the mean of the GZMA and PRF1 expression levels.^45^ The R package ‘ESTIMATE’ (version 2.0.0)^46^ was used to calculate the stromal score, immune score, ESTIMATE score and tumour purity. The stromal score and immune score were used to predict the fraction of stromal cells and the infiltration level of immune cells by expression data in tumour samples. These two scores formed the basis for calculating the ESTIMATE score. Tumour purity was defined as the proportion of tumour cells in a solid tumour sample, which can be inferred from the ESTIMATE score in this study.

### 2.3 Gene set enrichment analysis

The gene set enrichment analysis (GSEA) as implemented in the R package clusterProfiler (version 3.4.1)^47^ was used to identify whether the immune cell types were over‐represented in the tumour microenvironment. The tumour infiltration levels of 28 immune cell types for each BRCA patient were quantified by a single analysis of the enrichment analysis of regenerated cells (ssGSEA)^48^ that implemented in the R package GSVA (version 1.24.0).^49^ For using GSEA and GSVA software, the collected gene set of 782 marker genes in 28 immune cell types was chosen as the reference gene sets. The normalized enrichment score (NES) that calculated from the ssGSEA was considered as the tumour‐infiltrating level and used to examine the enrichment analysis results of 28 immune cell types.

### 2.4 Co‐expression network construction

The expression data profile of 782 immune marker genes was used to construct a gene co‐expression network for exploring the phenotype‐related immune genes and their interactions by using a weighted gene co‐expression network analysis (WGCNA) ^50^ that implemented in the R package WGCNA (version 1.24.0).^51^

### 2.5 Statistical analysis

Survival curves were estimated by using the Kaplan‐Meier method, and the differences between survival distributions were assessed by the two‐sided log‐rank test. The univariable survival analyses were performed by using Cox proportional hazards regression as implemented in R package survival (version 2.39‐5). The glmQLFTest that implemented in R package edgeR was used to identify the differentially expressed genes (DEGs).^52^, ^53^, ^54^ The KEGG pathway and immune system process enrichment analysis of these DEGs were performed and visualized by using the Cytoscape software (version 3.6.1) with the ClueGO (version 2.3.5).^55^ The GO biological process enrichment analysis was performed and visualized by the functional annotation tool enrichDAVID that implemented in R package clusterProfiler (version 3.4.1).^47^ All statistical analysis was performed in R 3.5.0. All of the statistical tests were two‐sided, and the differences with P‐values less than 0.05 were considered as statistically significant.

## 3 RESULTS

### 3.1 The immune infiltration differences between two BRCA subtypes

**Figure 1:**
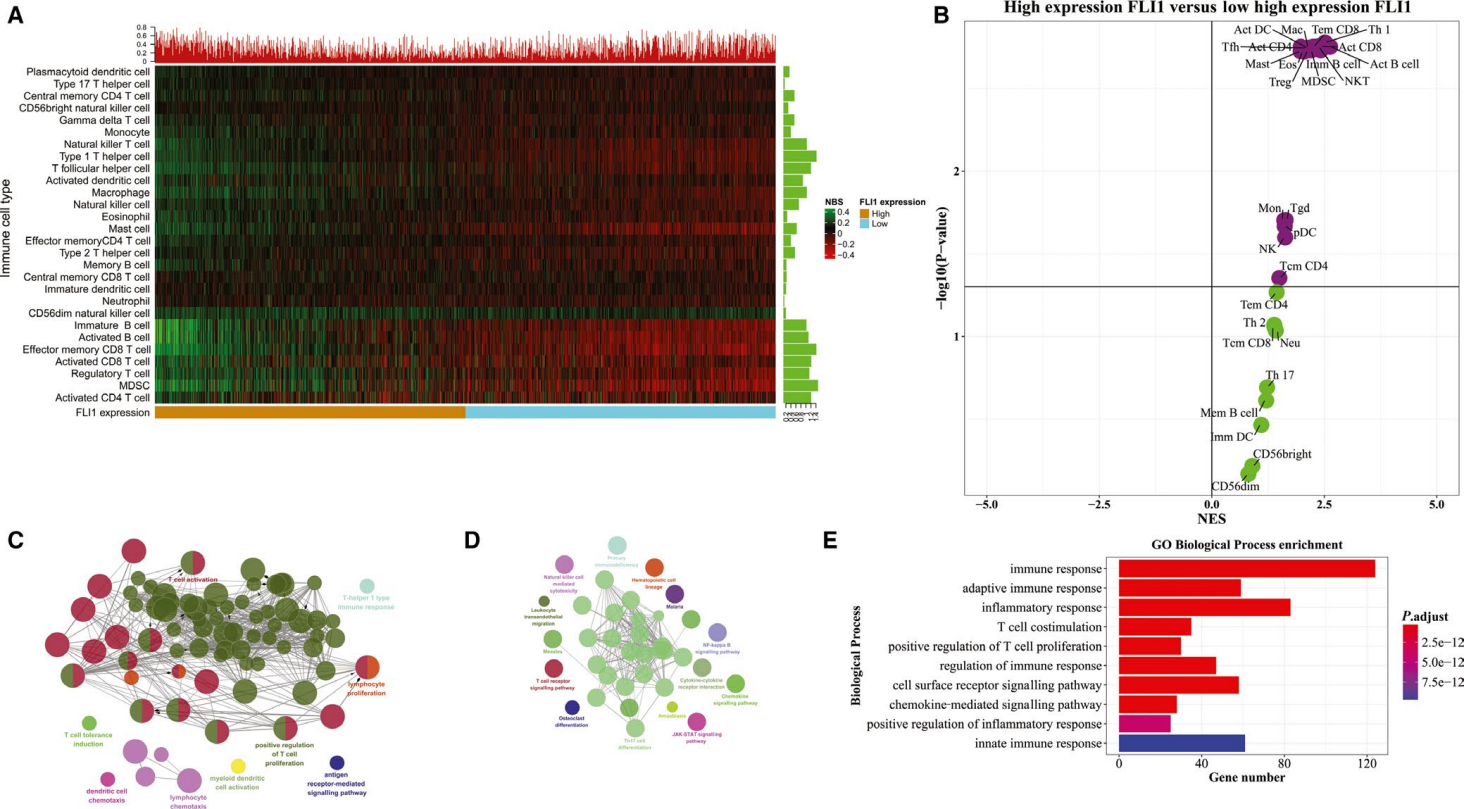
The immune infiltrate profile of BRCA. A, Heat map of 1095 breast cancer samples by using the ssGSEA scores from 28 immune cell types. Samples were arranged along the rows by two subtypes. Red‐green colour scale reflected magnitude. The barplot at the top indicated the percentages of 28 immune cell types which were significant. The barplot on the left indicated the percentages of BRCA samples which were significant. B, Volcano plot for the high FLI1 subtype that calculated from the GSEA when compared with the low FLI1 subtype. The visualization of (C) enriched immune system processes and (D) KEGG enriched pathways of the DEGs by using ClueGO (*P*‐value < .05). E, Top 10 statistically enriched biological processes by the DEGs

The tumour infiltration levels of 28 immune cell types for 1095 BRCA patients were quantified by using ssGSEA. A heat map was plotted to depict a more comprehensive picture of the immune infiltration landscape for breast cancer (Figure 1A). Based on median FLI1 expression level, the BRCA patients were classified into the high expression FLI1 subtype and low expression FLI1 subtype. In Figure 1A, 1095 BRCA patient samples were arranged along the row by the FLI1 expression level of patients, and 28 immune cell types were ordered along the column by clustering. Interestingly, samples with high FLI1 expression levels had high immune infiltration profiles in most regions. In contrast, samples with relatively low levels of FLI1 expression illustrated the low immune infiltration profiles in most regions.

Then, we compared the gene expression profile of the high expression FLI1 subtype samples with the low expression FLI1 subtype samples by the GSEA analysis to study the different TILs in these two subtypes of breast cancer. The NES scores and P‐values that generated by the GSEA enrichment results for the enriched and depleted immune cell types in two BRCA subtypes were visualized by the volcano plot Figure 1B. Then, the immune cell types were considered significantly enriched or depleted if the P‐value was less than 0.05. In the GSEA enrichment results (Figure 1B), we observed that the type 1 T helper cell, activated CD8^+^ T cell, natural killer T cell, activated B cell, activated CD4^+^ T cell, effector memory CD8^+^ T cell, activated dendritic cell, macrophage, T follicular helper cell, immature B cell, MDSC, regulatory T cell, mast cell, eosinophil, gamma delta T cell, monocyte, plasmacytoid dendritic cell, natural killer cell and central memory CD4^+^ T cell were significantly enriched in the high expression FLI1 subtype of breast cancer cohort. In summary, the GSEA analysis results implied that the high expression FLI1 subtype of BRCA was more associated with the enriched TILs.

In an effort to provide additional evidence to support the initial observation, the read count data set was also downloaded for BRCA samples for investigating the DEGs between the high expression FLI1 subtype and low expression FLI1 subtype. Finally, 955 up‐regulated DEGs were identified by using the false discovery rate (FDR) <0.05 and log 2 (fold change) more than 1.0 as the cut‐off. To annotate the function of these DEGs, all of these DEGs were used to perform the GO biological process enrichment analysis, KEGG pathway enrichment analysis and immune system process enrichment analysis, respectively. The GO biological process enrichment analysis of these 955 DEGs indicated that they were significantly enriched in many immune‐related biological processes, such as immune response, adaptive immune response and innate immune response (Figure 1C‐E).

Network visualization based on the immune system process enrichment analysis demonstrated that these DEGs were significantly enriched in 9 terms (Figure 1C), such as T cell activation, lymphocyte proliferation, positive regulation of T cell proliferation and lymphocyte chemotaxis. Network visualization based on KEGG pathway enrichment analysis was shown in Figure 1D. From this figure, we observed that these DEGs were commonly enriched in the immune‐related pathways, such as Th17 cell differentiation, natural killer cell‐mediated cytotoxicity and T cell receptor signalling pathway.

### 3.2 Correlations between FLI1 and immune‐related profiles

**Figure 2:**
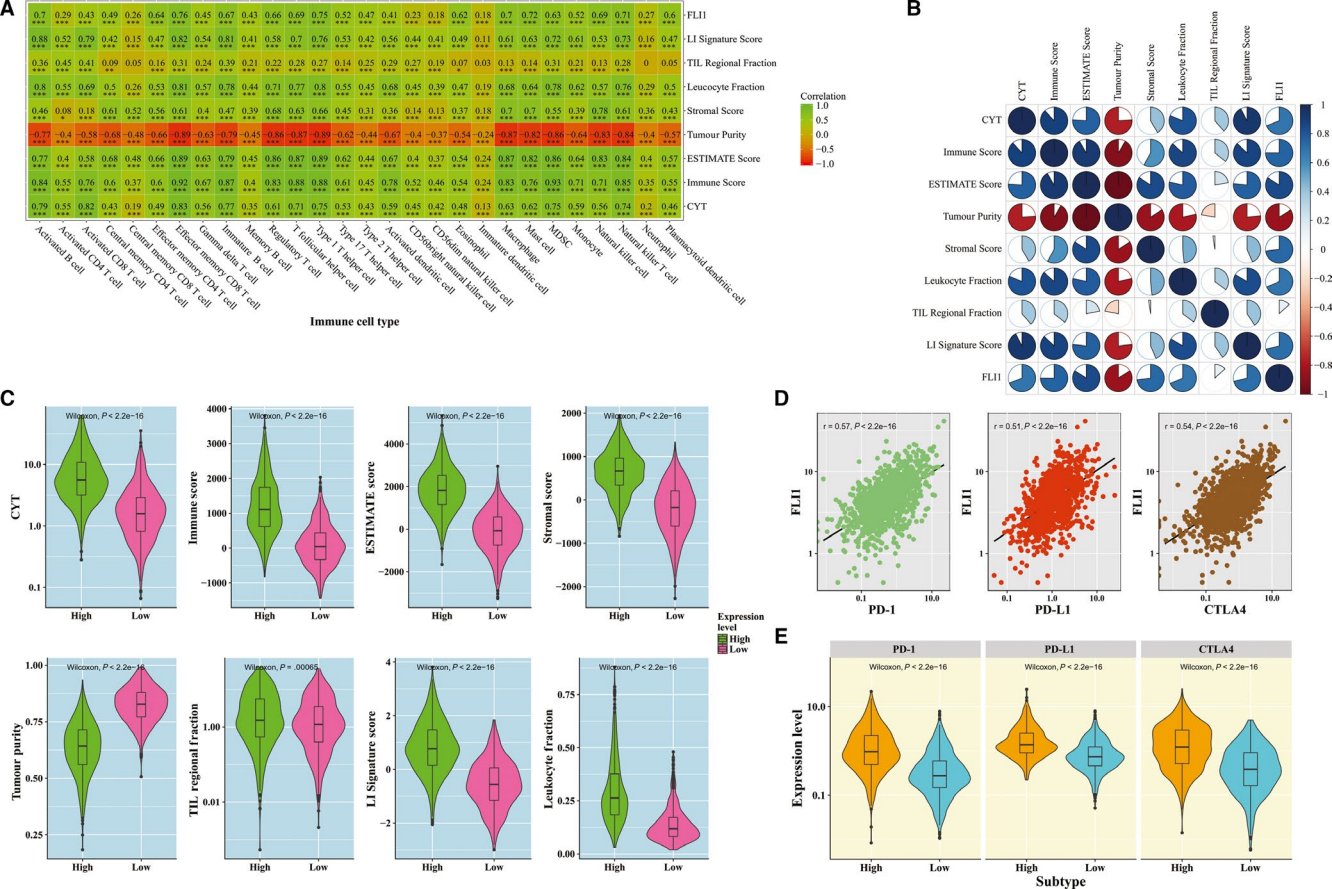
The relationship between the FLI1 and the immune‐related features in BRCA. A, Spearman's correlation between the ssGSEA scores of 28 immune cell types and the FLI1 expression level, LI signature score, TIL regional fraction, leucocyte fraction, stromal score, tumour purity, ESTIMATE score, immune score and CYT. Statistical significance at the level of null ≥ 0.05, * <0.05, ** <0.01 and *** <0.001. B, Spearman's correlation between the FLI1 expression level, LI signature score, TIL regional fraction, leucocyte fraction, stromal score, tumour purity, ESTIMATE score, immune score and CYT. The correlation coefficients were represented by red‐blue colour scale on the left. C, The violin plots of the CYT, immune score, ESTIMATE score, stromal score, tumour purity, TIL regional fraction, LI signature score and leucocyte fraction for two BRCA subtypes. D, Spearman's correlation between FLI1, PD‐1, PD‐L1 and CTLA4 expression level. E, The violin plots of the PD‐1, PD‐L1 and CTLA4 expression level for two BRCA subtypes

To identify whether the immune infiltration levels of 28 immune cell types were associated with FLI1 expression level in BRCA, the Spearman correlation tests were applied in 1095 BRCA samples between them (Figure 2A). As shown in Figure 2A, strong or moderate positive Spearman's correlations between the FLI1 expression level and ssGSEA scores were observed in most of the immune cell types. The Spearman correlations between FLI1 and effector memory CD8^+^ T cell, type 1 T helper cell and mast cell were 0.76, 0.75 and 0.72, respectively, which were the top three highest Spearman's correlations in this study. We also observed the weak Spearman correlations between FLI1 expression level and activated CD4^+^ T cell, central memory CD8^+^ T cell, CD56bright natural killer cell, CD56dim natural killer cell and immature dendritic cell and neutrophil. All the Spearman correlations were statistically significant according the statistical tests. These results indicated that FLI1 expression level in BRCA samples was associated with immune infiltrating levels.

Next, we calculated the Spearman correlation coefficients in 1095 BRCA patients between LI signature score, TIL regional fraction, leucocyte fraction, CYT, tumour purity, ESTIMATE score, immune score, stromal score and immune infiltrating levels of 28 immune subpopulations, and displayed the results in Figure 2A. Interestingly, we observed moderate or strong significant associations in most of them, which were similar to the association results of FLI1. We also found that most of the Spearman correlations between these features were moderate or strong in 1095 BRCA patients (Figure 2B).

The differences between the high and low expression FLI1 subtypes in the immune infiltrating levels of 28 immune cell subpopulations, CYT, immune score, ESTIMATE score, stromal score, tumour purity, TIL regional fraction, LI signature score and leucocyte fraction were also investigated in the BRCA patients (Figure S1 and Figure 2C). Except for the tumour purity, the average values of the high expression FLI1 subtype were significantly higher than those in the low expression FLI1 subtype for of all the other features. These results supported the conclusion that the FLI1 seemed to play an important role in immune systems, which needed more research in our future work.

Immune checkpoints are critical modulators in the immune system, allowing the initiation of a productive immune response and preventing the onset of autoimmunity. Among these immune checkpoints, PD‐1, PD‐L1 and CTLA‐4 were three important immune checkpoints. Here, we wanted to investigate whether the expression level of FLI1 in BRCA was associated with PD‐1, PD‐L1 and CTLA4. For doing this, we calculated the Spearman correlation coefficients between the expression level of FLI1 and three immune checkpoints. As illustrated in Figure 2D, the expression level of FLI1 was significantly positively correlated with the expression level of PD‐1, PD‐L1 and CTLA4. The expression levels of PD‐1, PD‐L1 and CTLA4 in the high FLI1 expression subtype were significantly higher than those of the low FLI1 expression subtype in the BRCA cohort (Figure 2E).

**Figure 3:**
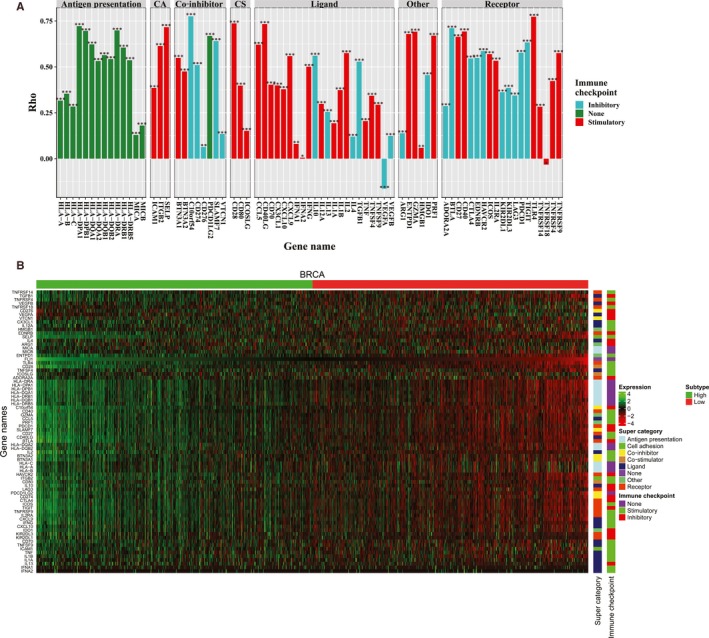
The relationship between the FLI1 and immunomodulators. A, Spearman's correlations between FLI1 expression levels and immunomodulators. Statistical significance at the level of null ≥ 0.05, *<0.05, **<0.01 and ***<0.001. B, The heat map for the expression levels of 75 immunomodulators. The immunomodulators were annotated by super category and immune checkpoint type

As numerous immunomodulator agonists were evaluated in clinical oncology, researchers found that immunomodulators were critical in cancer immunotherapy.^56^ To advance this research, it is needed to investigate the association between their expression levels and FLI1 expression level in BRCA. Associations between immunomodulator expression levels and FLI1 expression level were evaluated by using Spearman's correlation (Figure 3A). Figure 3A demonstrated that most of the immunomodulator expression levels had the moderate or strong positively correlations with FLI1 expression level. Among immunomodulators under investigation for cancer therapy, most of the immunomodulators demonstrated that the high expression levels in the high expression FLI1 subtype via a comparison against the low expression FLI1 subtype in BRCA. In summary, we had observed close associations between FLI1 and immunomodulators in BRCA.

### 3.3 Gene co‐expression network analysis

**Figure 4:**
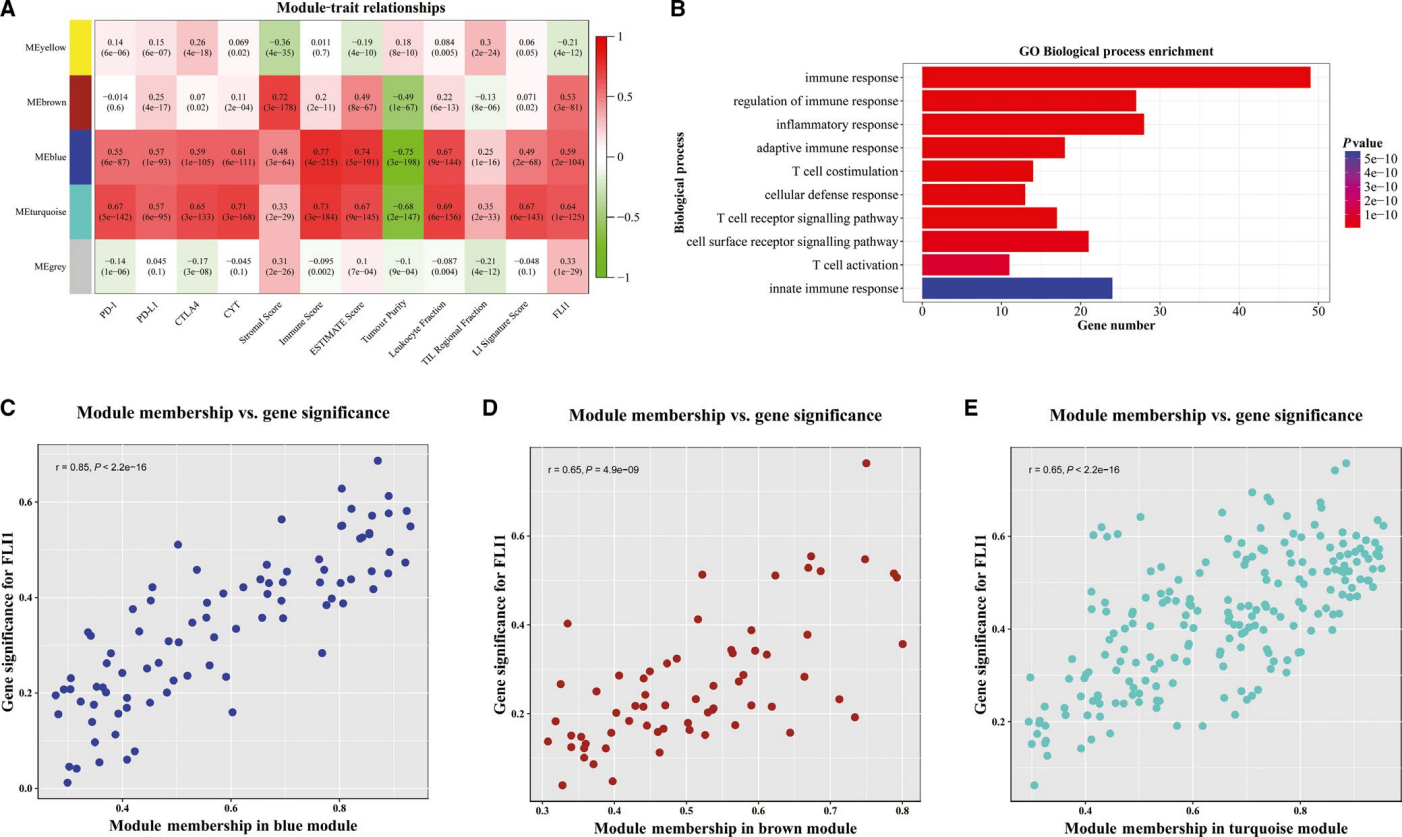
Identification of modules associated with the immune‐related features in BRCA by WGCNA. A, Heat map of the correlation between module eigengenes and immune‐related features in BRCA. B, Top 10 statistically enriched biological processes of turquoise module genes. The scatter plots between gene significance for FLI1 and module membership in (C) blue module, (D) brown module and (E) turquoise module. Each point corresponded to a gene in the module.

The WGCNA was widely used to explore modules of highly co‐expressed genes and explore the associations between gene sets and biological features. In this study, based on WGCNA algorithm, we wanted to provide additional evidence to support the associations between FLI1 and immune‐related features and to investigate which immune genes were more associated with FLI1 in BRCA. For doing these, the expression values of 782 immune genes in 1095 BRCA samples were used to construct the co‐expression network by WGCNA algorithm. The BRCA samples with FLI1 expression level, PD‐1 expression level, PD‐L1 expression level, CTLA expression level, CYT, tumour purity, ESTIMATE score, immune score, leucocyte fraction, TIL regional fraction and LI signature score were included in co‐expression analysis. The power of beta was selected as the soft thresholding to ensure a scale‐free network. A total of five distinct gene co‐expression modules were identified via the average linkage hierarchical clustering. 63, 65, 89, 207 and 348 co‐expression genes were included in the modules of yellow, brown, blue, turquoise and grey, respectively. The correlation between module eigengenes and FLI1 expression level, PD‐1 expression level, PD‐L1 expression level, CTLA expression level, CYT, tumour purity, ESTIMATE score, immune score, leucocyte fraction, TIL regional fraction and LI signature score of BRCA patients were identified (Figure 4A). Similar to the correlation results of immune‐related traits, including PD‐1, PD‐L1, CTLA, immune score, leucocyte fraction and LI signature score, FLI1 was significantly associated with blue module and turquoise module. These results may indicate that FLI1 expression level was correlated with immune‐related genes, like the previously described traits. In addition, we also found that the brown module showed high association with FLI1.

Among the identified modules, the turquoise module was found to have the highest association with FLI1. Based on this, the turquoise module was identified as the significant module, which was extracted for further analysis. We also found that the brown module and blue module shown high correlation with FLI1. To biologically characterize the genes in the turquoise module, we applied the DAVID tool to classify these genes and observed several GO biological process enrichment results in these three modules. According to GO biological process enrichment analysis (Figure 4B and Figure S2), our results demonstrated that the genes in turquoise module and blue module were mainly enriched in immune response, regulation of immune response, adaptive immune response and so on. The brown module showed no statistical significance for GO biological process enrichment. The scatter plots of gene significance (GS) for FLI1 versus module membership (MM) in the blue module, brown module and turquoise module were also plotted (Figure 4C‐E). The blue module, brown module and turquoise module exhibited significant correlation with FLI1 expression level in BRCA samples. Therefore, the FLI1 was considered to be significantly associated with blue module, brown module and turquoise module in breast cancer patients, which should be further investigated to understand the association between FLI1 expression level and immune gene expression level.

### 3.4 Prognostic significance of FLI1 in BRCA

**Figure 5:**
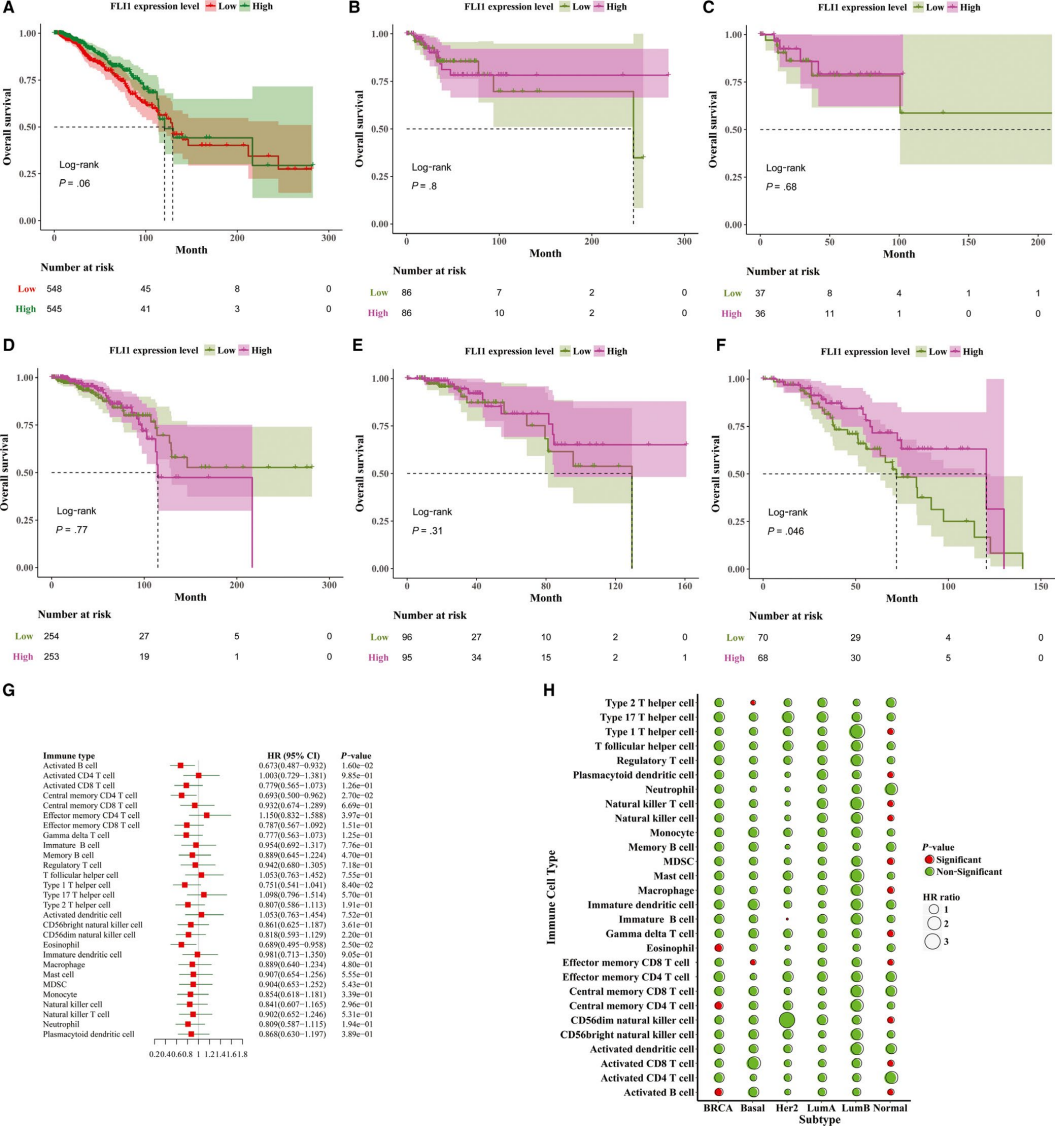
Prognostic significance of FLI1 in BRCA. Kaplan‐Meier survival curves by high and low FLI1 expression level for (A) BRCA (B) Basal (C) Her2 (D) Lum A (E) Lum B and (F) normal patients. G, Forest plot visualizing hazard ratios (HRs) with 95% CI and P‐values of 28 immune cell types in 1095 breast cancer patients. HR with 95% CI and P‐values was determined by univariate Cox proportional hazards regression analysis. H, Bubble plot for the hazard ratios and P‐values of 28 immune cell types in BRCA, Basal, Her2, Lum A, Lum B and normal patients

Considering the important immune role played by the FLI1 in BRCA patients, we want to investigate the association between the FLI1 expression level and prognosis of BRCA patients by the survival analysis and univariable Cox regression analysis. Overall survival differences between the low expression FLI1 subtype and the high expression FLI1 subtype were assessed by the Kaplan‐Meier estimate, and compared by using the log‐rank test. Patients with the high expression FLI1 subtype had higher median overall survival time than those of the low expression FLI1 subtype. However, the difference was not significant (129 months versus 121 months; log‐rank test *P*‐value = .06) (Figure 5A). The associations between the FLI1 expression level and the overall survival time in the five subtypes of breast cancer were also assessed by performing the same analysis (Figure 5B‐F). The patients in each subtype were classified into the high expression FLI1 group and the low expression FLI1 group by using the median FLI1 expression level in this subtype as the cut‐off point. Survival differences between the high FLI1 expression group and low FLI1 expression group in each subtype were assessed by the Kaplan‐Meier estimate and compared by using the log‐rank test. It was noteworthy that patients in the high FLI1 expression group had significantly longer median overall survival than those in the low FLI1 expression group in the normal subtype (124 months versus 72 months; log‐rank test, *P*‐value = .046) (Figure 5F). However, the associations of the FLI1 expression level with overall survival were not significant in the other four BRCA subtypes.

We next want to investigate whether the TILs of each patient were a prognostic factor in BRCA patient's overall survival time. For doing this, all the BRCA patients and the BRCA patients in each subtype were classified into two groups by using median ssGSEA scores as the cut‐off point, separately. The ssGSEA scores of each patient were considered as continuous variables in the univariable Cox regression analysis. By subjecting the ssGSEA scores of 28 immune cell types to univariable Cox regression analysis, we found that the high ssGSEA scores of activated B cells, central memory CD4^+^ T cells and eosinophils were significantly correlated with longer overall survival time in all BRCA patients (Figure 5G). For patients in the Basal subtype and Her2 subtype, the only one significant association was found between the immune infiltrates and overall survival time (Figure 5H). The univariable Cox analysis showed that the increased tumour‐infiltrating levels of type 1 T helper cells, plasmacytoid dendritic cells, natural killer cells, natural killer T cells, MDSCs, macrophages, gamma delta T cells, effector memory CD8^+^ T cells, CD56dim natural killer cells, activated CD8^+^ T cells and activated B cells were significantly associated with good prognosis in the BRCA normal subtype (Figure 5H). However, no significant association was found between the immune infiltrates and overall survival time in Lum A subtype and Lum B subtype (Figure 5H).

## 4 DISCUSSION

The knowledge of protein 3D (three‐dimensional) structures or their complexes with ligands is vitally important for rational drug design. Although X‐ray crystallography is a powerful tool in determining these structures, it is time‐consuming and expensive, and not all proteins can be successfully crystallized. Membrane proteins are difficult to crystallize and most of them will not dissolve in normal solvents. Therefore, so far very few membrane protein structures have been determined. NMR is indeed a very powerful tool in determining the 3D structures of membrane proteins,^57^, ^58^, ^59^, ^60^, ^61^ but it is also time‐consuming and costly. To acquire the structural information in a timely manner, a series of 3D protein structures have been developed by means of structural bioinformatics tools.^62^ Meanwhile, facing the explosive growth of biological sequences discovered in the post‐genomic age, to timely use them for drug development, a lot of important sequence‐based information, such as PTM (post‐translational modification) sites in proteins, have been successfully predicted.^63^, ^64^, ^65^ Actually, the rapid development in sequential bioinformatics and structural bioinformatics have driven the medicinal chemistry undergoing an unprecedented revolution,^66^ in which the computational biology has played increasingly important roles in stimulating the development of finding novel drugs. In view of this, the computational (or in silico) methods were also utilized in the current study.

With the explosive growth of biological sequences in the post‐genomic era, one of the most important but also most difficult problems in computational biology is how to express a biological sequence with a discrete model or a vector, yet still keep considerable sequence‐order information or key pattern characteristic. This is because all the existing machine‐learning algorithms (such as ‘optimization’ algorithm,^67^ ‘covariance discriminant’ or ‘CD’ algorithm,^68^, ^69^ ‘nearest neighbour’ or ‘NN’ algorithm,^70^ and ‘support vector machine’ or ‘SVM’ algorithm ^70^, ^71^) can only handle vectors as elaborated in a comprehensive review.^66^ However, a vector defined in a discrete model may completely lose all the sequence‐pattern information. To avoid completely losing the sequence‐pattern information for proteins, the pseudo amino acid composition ^72^ or PseAAC ^73^ was proposed. Ever since the concept of Chou's PseAAC was proposed, it has been widely used in nearly all the areas of computational proteomics.^65^, ^74^, ^75^, ^76^ Because it has been widely and increasingly used, four powerful open‐access softwares, called ‘PseAAC’,^77^ ‘PseAAC‐Builder’,^78^ ‘propy’ ^79^ and ‘PseAAC‐General’,^80^ were established: the former three are for generating various modes of Chou's special PseAAC,^81^ while the 4th one for those of Chou's general PseAAC,^29^ including not only all the special modes of feature vectors for proteins but also the higher level feature vectors such as “Functional Domain” mode, “Gene Ontology” mode, and “Sequential Evolution” or “PSSM” mode. Encouraged by the successes of using PseAAC to deal with protein/peptide sequences, the concept of PseKNC (pseudo K‐tuple nucleotide composition) ^82^ was developed for generating various feature vectors for DNA/RNA sequences ^83^, ^84^, ^85^ that have proved very useful as well. Particularly, in 2015 a very powerful web server called ‘Pse‐in‐One’ ^86^ and its updated version ‘Pse‐in‐One2.0’ ^87^ have been established that can be used to generate any desired feature vectors for protein/peptide and DNA/RNA sequences according to the need of users’ studies.^87^

As mentioned in previous studies, the high levels of FLI1 expression were found in T cells, B cells and several other types of immune cells, suggesting that the importance of FLI1 in the immune system.^12^, ^14^, ^88^ In this study, we had shown for the first time that the expression level of FLI1 in BRCA samples was associated with the immune infiltration levels of many immune cell types and many immune‐related features, including CYT, tumour purity, ESTIMATE score, immune score, stromal score and immunomodulators.

By using the median expression level of FLI1 as the cut‐off point, the BRCA patients were divided into a high expression FLI1 subtype and a low expression FLI1 subtype. Compared with the low expression FLI1 subtype, patients in the high expression FLI1 subtype had stronger immune cell infiltration and anti‐tumour immune activities. For example, high expression FLI1 subtype had high levels of T cell and B cell infiltration. When we used ssGSEA to calculate the proportions of 28 immune cell types in BRCA, we found that the infiltration levels of all immune cell types tended to be significantly higher in high expression FLI1 subtype than in low expression FLI1 subtype (*P*‐value < .05, Wilcoxon's test) (Figure S1). These results confirmed the elevated anti‐tumour immune activity in the high expression FLI1 subtype. We examined the expression levels of PD‐L1 in the two BRCA subtypes and found that the high expression FLI1 subtype had the higher PD‐L1 expression levels when compared with the low expression FLI1 subtype, and the difference between them was significant (*P*‐value < 2.20E‐16, Wilcoxon's test). These results indicated that the high expression FLI1 subtype might better respond to anti‐PD‐L1 immunotherapy than the low expression FLI1 subtype, as PD‐L1 expression level tended to be positively associated with immunotherapeutic responsiveness.^89^

In order to further understand the possible biological functions of the high expression FLI1 in BRCA, the DEGs between the high expression FLI1 subtype and the low expression FLI1 subtype were identified and functional enrichment analysis was performed. The DEGs between these two subtypes were significantly enriched in many immune‐related biological processes and KEGG pathways by performing functional enrichment analysis. The analysis results indicated that the FLI1 was associated with immune‐related profiles.

**Figure 6:**
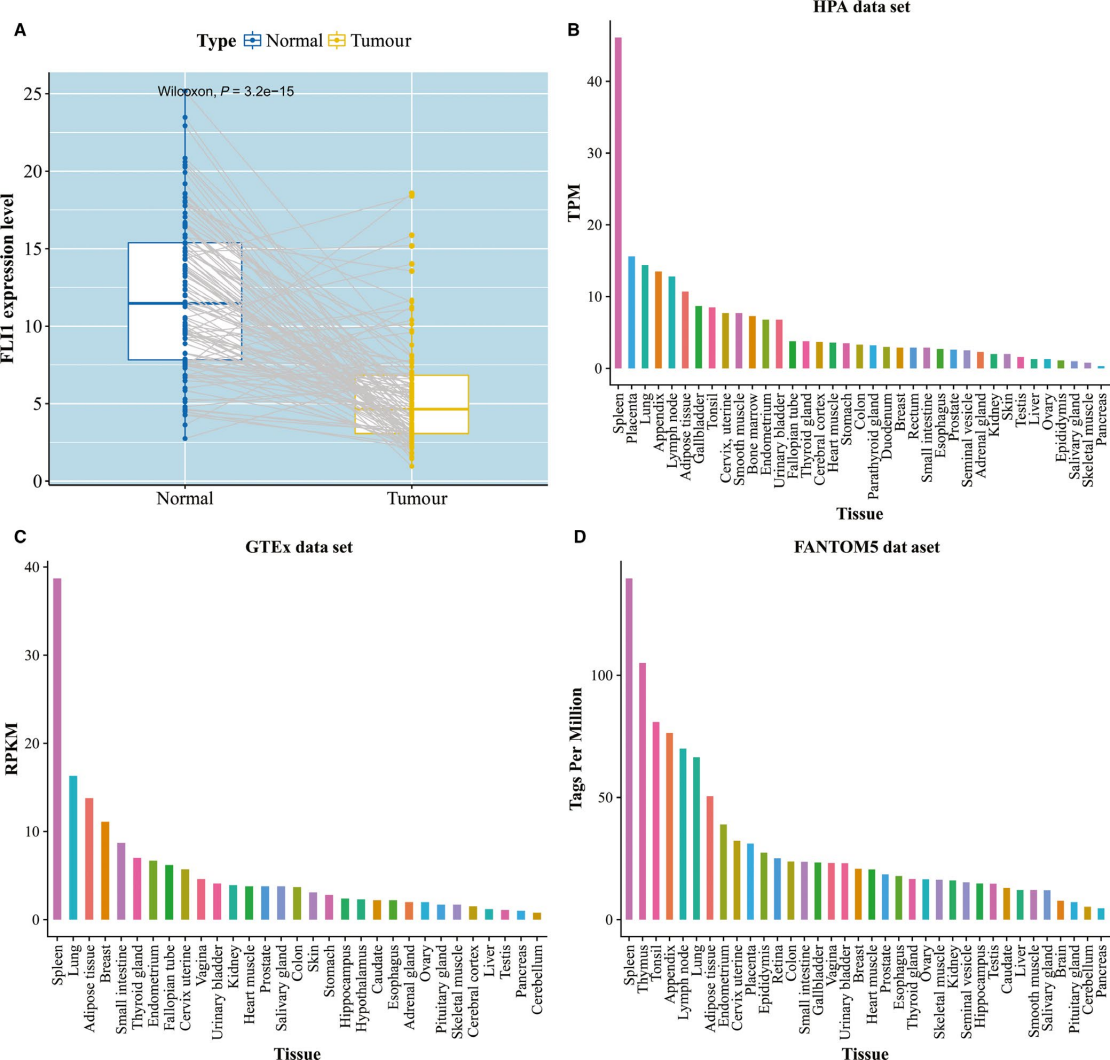
The evaluation of FLI1 expression level. A, Pairwise comparison of FLI1 expression level by normal and tumour tissues. The FLI1 expression level in human different tissues from (B) HPA data set, (C) GTEx data set and (D) FANTOM5 data set

We also downloaded the transcriptional profiles of 113 paired normal samples for BRCA and compared the FLI1 expression levels of the normal samples with their paired tumour samples. We found that the FLI1 expression levels of the normal samples were significantly increased when compared with those of their paired tumour samples (Figure 6A), which was consistent with previous work.^88^ To evaluate the FLI1 expression levels in human different tissues, we analysed FLI1 in the Human Protein Atlas (HPA).^90^ The tissue specific expression in all analysed tissues on FLI1 expression levels was plotted from three different RNA expression data sets: including 37 tissues from HPA data set, 31 tissues from Genotype‐Tissue Expression (GTEx) consortium and 35 tissues FANTOM5 consortium (Figure 6B‐D). These figures illustrated that the FLI1 was simultaneously elevated in spleen, when compared to all other analysed tissues. Spleen was believed to play an important role in the immune response and systemic regulation of innate and adaptive immunity.^91^ This indicated that FLI1 may be an important gene in immune system. These observations further confirmed the results that the FLI1 expression level was associated with immune‐related profiles.

The association of the FLI1 expression level with survival time was also investigated. However, no significant survival difference was found by the log‐rank test. By conducting the univariate Cox regression analysis on the tumour‐infiltrating levels of 28 immune cell types, these results showed that the increased tumour‐infiltrating levels of activated B cells, central memory CD4^+^ T cells, and eosinophils were significantly associated with longer overall survival time in the BRCA patients. When extending the same analysis to five subtypes of BRCA patients, we found that the increased tumour‐infiltrating levels of 11 immune cell types were significantly associated with longer overall survival time in the normal subtype of BRCA patients, which was more than other four subtypes. These results indicated that the normal subtype of BRCA was more associated with the immune cell types than the others, and this may explain why the high expression FLI1 subtype of normal subtype was significantly associated with overall survival time.

In this study, a simple and readily adapted method was applied for inferring immune infiltration levels of different immune cell types in tumours. There were several advantages in this study. First, using a set of immune genes for decomposing of immune cell types was more robust than using only one gene, as several genes were expressed in many different immune cell types. Second, assessing the relative expression changes of a set of genes with the expression of all other genes in one sample by GSEA was less sensitive to noise. Third, using the BRCA samples in TCGA may be more advantageous than using the BRCA samples from other data sets, as more comprehensive data set about the immune infiltration levels can be obtained in the TCGA data set.

In summary, the FLI1 expression level was found to be associated with immune infiltration profiles in BRCA patients. Immunotherapy for breast cancer is an active field of investigation, and the higher immunogenicity exhibited by the high expression FLI1 subtype compared to the low expression FLI1 subtype indicated that immunotherapy could be a viable option for the patients with the high FLI1 expression level. With the increased understanding of the tumour immune microenvironment played an important role in tumour therapy and patient prognosis, these findings may play a critical role for immunotherapy.

Using graphic approaches to study biological and medical systems can provide an intuitive vision and useful insights for helping analyse complicated relations therein as shown by the eight master pieces of pioneering papers from the then Chairman of Nobel Prize Committee Sture Forsen ^92^, ^93^, ^94^, ^95^, ^96^, ^97^, ^98^, ^99^ and many follow‐up papers.^100^, ^101^, ^102^, ^103^ As shown in a series of recent publications ^104^, ^105^, ^106^, ^107^, ^108^, ^109^, ^110^, ^111^, ^112^, ^113^, ^114^, ^115^, ^116^, ^117^ in demonstrating new findings or approaches, user‐friendly and publicly accessible web servers will significantly enhance their impacts,^66^ driving medicinal chemistry into an unprecedented revolution,^76^ we shall make efforts in our future work to provide a web server to display the findings that can be manipulated by users according to their need.

## CONFLICT OF INTEREST

The authors declare no conflict of interest.

## AUTHORS’ CONTRIBUTIONS

YY, YP, DS and QL performed data analyses and helped prepare for the manuscript. LY, SW, WY and YZ conceived the research and designed the analyses strategies. CY, YC and YW wrote the manuscript. All the authors read and approved the final manuscript.

## Supporting information

Fig S1

Fig S2

Supplementary Material

## Data Availability

The data in the current study are available from the corresponding authors on reasonable request.
