# Supplementary figures and images for: Characterization of the relationship between FLI1 and immune infiltrate level in tumour immune microenvironment for breast cancer
Source: J Cell Mol Med. 2020 Apr 5;24(10):5501–14. doi: 10.1111/jcmm.15205 (PMC7214163; doi:10.1111/jcmm.15205)

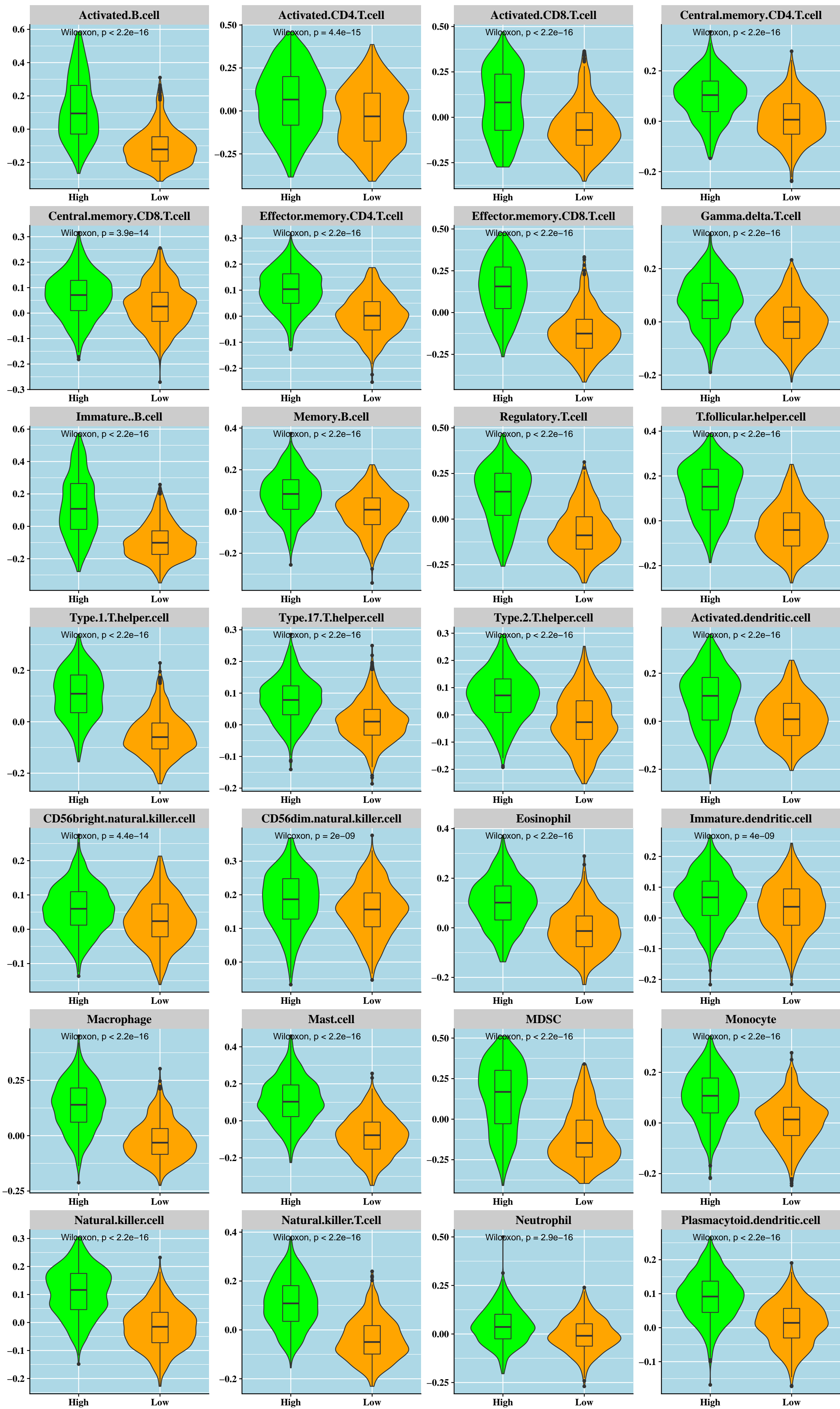

Supplement: Supplementary file 1 — Fig S1 [file JCMM-24-5501-s001.pdf]

## GO Biological Process enrichment

Biological Process

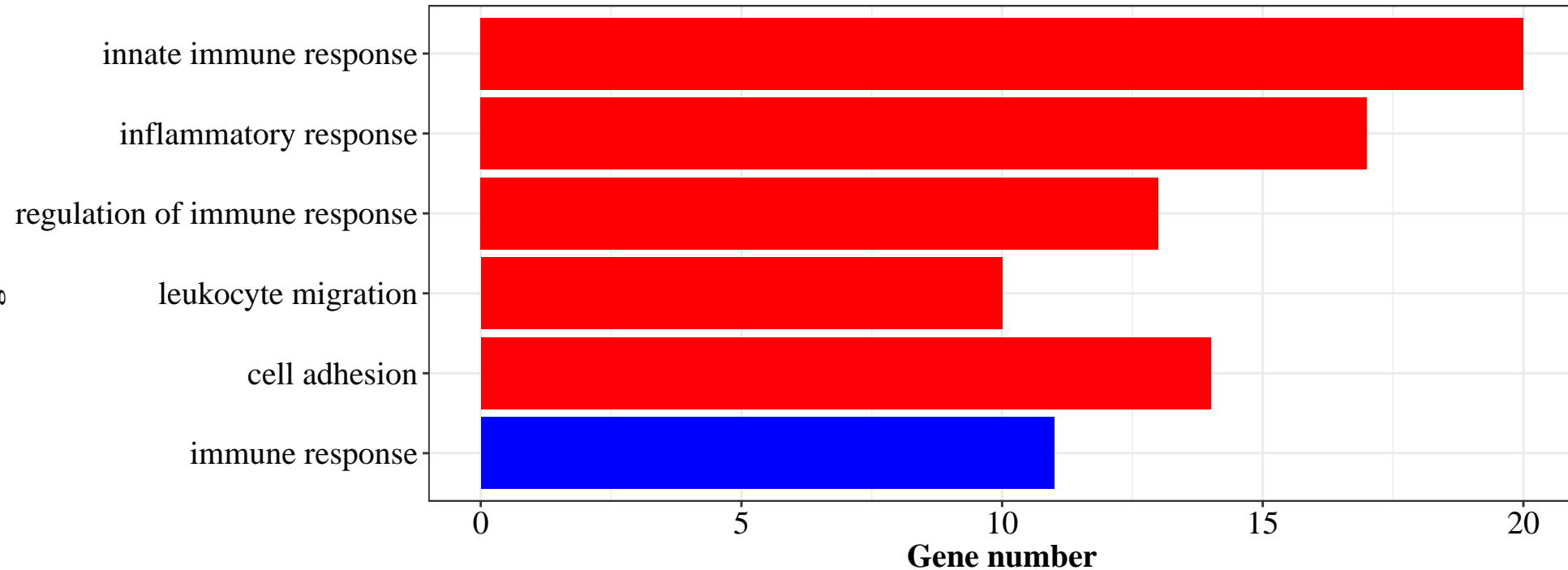

Supplement: Supplementary file 2 — Fig S2 [file JCMM-24-5501-s002.pdf]
