# Supplementary material for: Characterization of the relationship between FLI1 and immune infiltrate level in tumour immune microenvironment for breast cancer
Source: J Cell Mol Med. 2020 Apr 5;24(10):5501–14. doi: 10.1111/jcmm.15205 (PMC7214163; doi:10.1111/jcmm.15205)
Supplement: Supplementary file 3 — Supplementary Material [file JCMM-24-5501-s003.docx]

**Supplementary Figure Legend**

**Fig. S1** The violin plots of the 28 immune cell types for the high expression and the low expression FLI1 subtype in BRCA patients.

**Fig. S2** GO biological process enrichment analysis of genes in blue module.
